# “We’re already doing this work”: ethical research with community-based organizations

**DOI:** 10.1186/s12874-022-01713-7

**Published:** 2022-09-02

**Authors:** Rebecca Fielding-Miller, Sarah Kim, Jeanette Bowles, Samantha Streuli, Peter Davidson

**Affiliations:** 1 University of California, 9500 Gilman Drive #0507, La Jolla, San Diego, CA 92093 USA; 2 San Diego, USA; 3 Centre On Drug Policy and Evaluation, Vancouver, Canada

**Keywords:** Public health, Community-based organizations, Research ethics, Research personnel

## Abstract

**Background:**

Public health research frequently relies on collaborations with community-based organizations, and these partnerships can be essential to the success of a project. However, while public health ethics and oversight policies have historically focused on ensuring that individual subjects are protected from unethical or unfair practices, there are few guidelines to protect the organizations which facilitate relationships with – and are frequently composed of – these same vulnerable populations. As universities, governments, and donors place a renewed emphasis on the need for community engaged research to address systematic drivers of health inequity, it is vital that the ways in which research is conducted does not uphold the same intersecting systems of gender, race, and class oppression which led to the very same health inequities of interest.

**Methods:**

To understand how traditional notions of public health research ethics might be expanded to encompass partnerships with organizations as well as individuals, we conducted qualitative interviews with 39 staff members (executive directors and frontline) at community-based organizations that primarily serve people who use drugs, Black men who have sex with men, and sex workers across the United States from January 2016 – August 2017. We also conducted 11 in-depth interviews with professional academic researchers with experience partnering with CBOs that serve similar populations. Transcripts were analyzed thematically using emergent codes and a priori codes derived from the Belmont Report.

**Results:**

The concepts of respect, beneficence, and justice are a starting point for collaboration with CBOs, but participants deepened them beyond traditional regulatory concepts to consider the ethics of relationships, care, and solidarity. These concepts could and should apply to the treatment of organizations that participate in research just as they apply to individual human subjects, although their implementation will differ when applied to CBOs vs individual human subjects.

**Conclusions:**

Academic-CBO partnerships are likely to be more successful for both academics and CBOs if academic researchers work to center individual-level relationship building that is mutually respectful and grounded in cultural humility. More support from academic institutions and ethical oversight entities can enable more ethically grounded relationships between academic researchers, academic institutions, and community based organizations.

## Introduction

Community engagement is a core tenet of public health practice and research. Health equity has always been a concern for many public health professionals; however the COVID-19 pandemic has led to increased visibility of disparate health outcomes for communities that have been made socially vulnerable by historical and current medical mistreatment, economic disinvestment, and discriminatory legal frameworks. In the United States these structural factors have most affected communities of color, with significantly worse effects for people who experience multiple forms of intersectional marginalization, such as gender and sexual minorities, people who use drugs, people with disabilities, and/or people experiencing homelessness (Poteat et al., [1]; Jashinsky et al., [2]). In the United States, many government entities have generated policy documents emphasizing the importance of partnering with affected communities when working to address these health disparities in COVID-19 and myriad other public health concerns (California Department of Public Health, [3]).

Community engaged research (CEnR) in public health takes many forms. Broadly defined, community engagement is “the process of working collaboratively with and through groups of people affiliated by geographic proximity, special interest, or similar situations to address issues affecting the well-being of those people.”(Principles of Community Engagement, [4]). This collaborative process is best understood as a spectrum rather than a single set of practices, ranging from a complete lack of community involvement, to sporadically informing the community about the research on one end, to community based participatory research (CBPR) and community led research initiatives on the other (Wallerstein and Duran, [5]; Key et al., [6]). The CBPR approach emphasizes power sharing and equitable collaboration between the researcher and the community (Wallerstein and Duran, [5]; Wallersetin and Duran, [7]). However, the vast majority of public health research in community settings does not involve CBPR collaborations, nor would this time- and resource-intensive approach necessarily be appropriate for many behavioral or biomedical studies as there is little institutional or research support for these approaches (Fregonese, [8]). Further, while CBPR techniques are particularly valuable (and achievable) when the “community” at the center of the research is a self-defined community where membership is clear to both insiders and outsiders and clear voices exist who can ‘speak for the community, CBPR becomes difficult or impossible where the ‘community’ (or ‘population’) has been defined externally around a shared behavior rather than a shared identity (Minkler and Wallerstein, [9]; Israel et al., [10]).

The “community” with which researchers partner in community engaged (CE) work can also manifest across a spectrum of formality, from a single individual who is an acknowledged (or designated) community leader, to formal agreements between academic institutions and community-based organizations (CBOs). Community-based organizations “are public or private not-for-profit resource hubs that provide specific services to the community or targeted population within the community” (Beste et al., [11]). Collaborations with CBOs can function as the primary means of community engagement (CE) in a study or they may be just one aspect of a multifaceted CEnR or CBPR project. These CBO-academic collaborations encompass a wide range of activities, from posting recruitment materials, to providing space for study activities, to formal fiscal arrangements to involve the CBO in recruitment, retention, or other study activities.

A substantial body of scholarship, law, and regulation has developed since the second world war which addresses the ethical issues involved in medical human subjects research (The Nuremberg Code, [12]; Association WM, [13]; The Belmont Report, [14]). This body is primarily grounded in the notion of *principlist* ethics, as best expressed by the Belmont Report (Hammersley, [15]) and codified in the United States federal regulations which govern the institutional review boards (IRBs) tasked with providing ethical oversight for academic research projects (Holm, [16]). The Belmont Report frames the primary ethical obligations of medical research around the principles of respect, beneficence (and its corollary, nonmaleficence), and justice. In practice, these typically manifest as respect for participant autonomy via informed consent, an attempt to maximize benefit to research participants and minimize harm, and attention to ensuring that the benefits of the research do not primarily accrue to one population while the risks primarily accrue to another (The Belmont Report, [14]). There have been significant critiques of this model over the years. Academics and communities have described many ways in which this top-down approach can misalign with both the practice and spirit of CBPR projects (Banks et al., [17]; Shore, 2008). Others have questioned the epistemology of the framework itself, suggesting that a positivist notion of the ‘objective’ researcher engaged in the search for pure truth is both inappropriate to the messy realities of research with human subjects, and elides very real issues with implicit white supremacy, imperialism, and cisgendered heteropatriarchy embedded in the history of medical research and bioethics (Douglas, [18]; Anderson, [19]; Holm, [16]). More pragmatically, the daily reality of conducting human subjects research, particularly projects that partner with communities or individuals that have been made vulnerable by structural forces, can often throw the difference between “wicked,” thorny ethical problems into contrast with the neater, premeditated “compliance” ethics which academic researchers carefully document for institutional review boards (Heimer, [20]).To counter these issues, other scholars have introduced the notion of ethics as a relational practice, considering care as a core component of working ethically with human subjects (Etherington, [21]; Ward and Gahagan, [22]).

Many researchers and ethicists have also voiced concern that the bioethical principles expressed in these laws and regulations, while well-considered for the purpose of protecting the rights and well-being of individual research participants, do not provide guidance in the conduct of research affecting entire communities (Brugge and Kole, [23]; Flicker et al., [24]; Mikesell et al., [25]). Likewise, regulatory mechanisms (such as IRBs) tend to focus on the risks and benefits that might accrue to individual study participants rather than the risk and benefits which might accrue to communities (Flicker et al., [24]; Shore, [26]; Banks et al., [17]; Participants in the Community Engagement and Consent Workshop, [27]).

The authors of the present study have all worked extensively with CBOs, most typically in research related to HIV, violence, health equity, or substance use, and we have all heard about and witnessed the frustrations CBOs have with researchers. As researchers, we have all trained in the protocols that Institutional Review Boards [IRBs] and the Belmont Report (1979) have set forth for protecting human subjects, however, we have all noted the striking lack of ethical safeguards for CBOs in the research process. In the present study, we sought to understand how traditional notions of public health research ethics might be expanded to encompass the work public health researchers frequently do in partnership with community-based organizations.

## Methods

### Researcher positionality statement

The authors recognize the power imbalance inherent in research relationships between academics and community partners, including our own relationships with the organizations and individual respondents involved in the present study. While researchers have been historically positioned as “unmarked” arbiters of objectivity, feminist and critical race theory approaches have identified the importance of researcher identity in the design, conduct, and interpretation of research (Haraway, [28]/1992; Hill Collins, [29]). RFM identifies as a white woman and was a postdoctoral fellow under the supervision of PD at the time these interviews were conducted and is now an Assistant Professor. JB identifies as a white woman and worked on this manuscript as a postdoctoral fellow under the supervision of PD. SK identifies as an Asian woman and worked on this manuscript as an undergraduate research assistant under the supervision of RFM. SS identifies as a non-binary white woman and is currently a postdoctoral fellow under the supervision of RFM. PD identifies as a white male and is an Associate Professor.

All authors have engaged in research partnerships with CBOs or have been employed by CBOs, and as such approach this work with a personal commitment to advancing equitable academic-CBO partnerships.

### Recruitment and study design

This study used a modified grounded theory approach based in perspectivism, an epistemological lens which acknowledges the multiple perspectives from which the researcher can approach and view a phenomenon as well as the critical need to incorporate the perspective of the community under study, and hence the importance of utilizing multiple methodologies to understand the phenomenon (Tebes, [30]).

We recruited leaders and frontline staff from community-based organizations (CBOs) across the country. We specifically sought out CBOs that serve Black or African American men who have sex with men (MSM), sex workers, and people who inject drugs. This decision was largely driven by our personal experience as researchers working in the fields of harm reduction and HIV prevention. These three communities are frequently labeled ‘high risk,’ or – more pointedly – “at high-risk for the spreading of HIV infection” and as such are often targeted by academic researchers who are seeking to make their project more attractive for funding from the National Institutes of Health (NIH) (Institute of Medicine, [31]). We also sought out professional academic researchers who had engaged in formal partnerships with CBOs, or who had substantial experience with community-engaged or participatory research.

We used several strategies to recruit participants: A ‘CBO Expert Group’ was formed comprising the executive directors of 6 community-based organizations with national reputations who provide social and/or health services to one or more groups of people who use drugs, people engaged in sex work, and Black men who have sex with men. CBO Expert Group members provided introductions to executive directors or other senior staff at other organizations serving the same populations. The first and last authors (RFM, PD) attended multiple conferences at which CBOs serving the three populations above were present and we either presented preliminary data from the present study, and/or placed advertisements in conference materials about the study. Finally, we asked study participants to suggest other individuals or organizations who they felt would be able to contribute to this study, and, where appropriate, to make email introductions to those people. Due to the nature of the complex power dynamics between CBOs and academic researchers (as well as early career and senior academic researchers) we only recruited study participants from organizations with which the authors had not worked in the past. In the few instances in which pre-existing relationships did exist between a CBO or academic and study team member, that study team member did not conduct the interview.

We designed the initial field guide with input from the CBO Expert Group. The field guide was semi-structured and was revised iteratively as interviewing progressed. The guide covered three main domains: (1)CBO staff were asked to narrate their decision making process when evaluating requests to collaborate with academic researchers – an approach based on ethnographic decision tree modeling (Gladwin, [32]), (2)CBO staff and academic researchers were asked to narrate stories of particularly good or bad collaborative experiences, and (3)CBO staff were asked to comment on the utility of the Belmont framework (emphasizing respect, beneficence, and justice) when considering ethical interactions between CBOs and academic researchers. Interviews were face to face and were conducted in private or semi-private spaces, most commonly at the participant’s workplace, a coffee shop or other semi-private space chosen by the participant. A smaller number of interviews were conducted by telephone or video call. All interviews were recorded and recordings were transcribed by a professional transcriptionist.

### Analysis

Analysis was an iterative process, following Creswell’s ‘spiral’ (Creswell and Poth, [33]). RFM and PD met regularly during the course of data collection to discuss emergent themes and to modify the field guide and sampling strategy as appropriate based on these. After RFM and PD agreed saturation had been reached within the CBO and academic subgroups, and across the sample as a whole, RFK, SK, and JB reviewed the transcripts and developed a codebook in discussion with PD Codes were a combination of emergent themes and concepts drawn from the Belmont report (i.e., respect, beneficence, justice). Transcripts were coded by RFM, SK, and JB, and the codebook was refined via iterative discussions with the full study team. All coding was done using MaxQDA software (VERBI Software, [34]). The research team met frequently to discuss coding and data synthesis and to resolve differences in transcript coding. We periodically presented initial findings at conferences (generally the same conferences from which we were recruiting) to engage in member-checking and gather feedback on our preliminary conclusions.

### Regulatory ethics

Respondents were given $50 to thank them for their time and expertise immediately after informed consent and before commencing the interview. The study’s ethical protocol was approved by the University of California, San Diego Institutional Review Board.

## Results

We conducted 39 in-depth interviews with the directors and frontline staff of 25 community-based organizations (CBOs), and 11 in-depth interviews with researchers who had previously collaborated with CBOs. Participants were recruited across 14 metropolitan areas in the United States and a wide range of organization types, including sex worker led advocacy groups, churches, halfway houses contracted with the federal bureau of prisons, syringe exchange programs with varying degrees of access to legal documentation, formal umbrella organizations with the resources to host national conferences, and community-based consultancies consisting of one to two individuals. Participants who worked as professional academic researchers were typically based at research intensive universities or had been previously based at a research-intensive university before transitioning to a think tank or consultancy model.

CBO and academic-research participants agreed that the concepts of respect, beneficence, and justice could and should apply to the treatment of organizations that participate in research just as they apply to individual human subjects. Although their implementation often differed when applied to CBO partnerships vs individual human subjects. The individuals we spoke with also embraced a broader meaning of these principles, aligning them more closely with a relational rather than purely regulatory or principlist framework.

When we conducted comparative analyses between professional academic researchers and CBO staff, we found that participants were in concordance on all major themes. Because our findings were the same across participant categories, our team made the deliberate decision to center CBO voices in the present manuscript. To that end, all illustrative quotes in the data presented below are from CBO staff.

### Respect

The vast majority of participants situated respect as the foundation to any successful, ethical partnership between academic researchers and CBOs. In the Belmont Report, respect is grounded in individual autonomy and is primarily operationalized through informed consent. In CBO staff conceptualization, respect is an ongoing practice, and participants repeatedly emphasized that respect was demonstrated by the researcher’s willingness to practice being in purposeful, individual level relationships with CBO staff and clients.

CBO participants framed respect as an active process that took place within relationships. While participants were aware that research relationships are formally made between institutions (i.e., a subcontract from a university to a CBO), academic researchers (and their study staff) were judged on their ability and willingness to act respectfully within individual interpersonal relationships. Several participants expressed frustration with ‘arrogant’ researchers, and others noted the ways in which academic research could replicate hierarchies of oppression and power, either within the academic study team and/or in the relationship between the researcher and the CBO:

*[P]eople* expect service providers to bend over backwards to accommodate their oh so precious project, you know… *So there’s an arrogance about--that they’re somehow imparting some gift to us, which is most of the time not the case*… *We’ve had research assistants … they just come chill in the drop-in center and it’s like, who are these people*? There’s just sort of this privilege to feel like you just walk into this space and observe… A lot of privilege. A lot of white men researchers. And I even see that dynamic too with white men researchers who either employ research assistants who are super new…who aren’t from the community… And … I have observed those dynamics with the PI’s before, where they’re just--like they’re a gift from God. And that the research assistants are their minions and there’s a weird lack of respect that I’ve observed.

-Former director of HIV and reproductive health NGO

Several organizations described engaging in official or unofficial vetting practices to determine if researchers were capable of being respectful of an organization and their clients. Some CBOs would require researchers to fill out application forms, both to determine project fit and to assert their intention to stand on an equal footing with the researcher. Others would encourage researchers to spend time volunteering before initiating a project together. This gave staff an opportunity to see how researchers would engage with clients and staff before committing to a prolonged project:

Whenever possible, we do our best to bring them with us. The first thing that someone who wants to work with us does, is observe us in action. They hang out in the drop-in center. They come with us to outreach. And we watch them. How do you interact with the people? Sometimes we might give them a little task at outreach. … “Hey, help us pass out or distribute some of this bread and pastry.” *How do you interact with the people, are you respectful?*

- Director, Harm Reduction Organization

For CBO staff, researchers could also demonstrate mutual respect through transparent communication and power sharing throughout the process of research conception, design, implementation, and dissemination. The precise details varied according to CBO mission and needs, but all participants emphasized that if the researcher’s approach to the partnership was characterized by cultural humility and a desire to meet CBO staff and clients where they were then the study was much more likely to be implemented smoothly. Many participants felt that they could evaluate the likelihood that a project would be a successful, mutually beneficial partnership based on the researcher’s initial question and their willingness or institutional ability to adapt it according to the CBOs needs.

*A big piece of it is the approach and does the conversation start with, … “I’m really interested in this question and I’m stuck and I feel like you have the ability to help me think through it,” […] that I think is super productive and a great way to start*. But if someone comes in and they’re like, “We have this great opportunity for [your CBO]. We’ll give you $1,000 to recruit ten people, or this is really a great opportunity for you to get involved in research,” or whatever, that’s not helpful. Then I just want to say, “That’s super and thank you and we have lots of opportunities, so we’re good”…. I think that community based organizations, for as difficult or problematic as we are, we do a lot of really good work and so for a researcher to approach the organization … showing some sort of respect or offering them some dignity, *this is what you need to do with people in general.*

*-MSM CBO Director*

### Beneficence

Participants agreed that researchers had an obligation to minimize the potential harm to an organization and maximize the possible benefits. Some CBOs shared stories of specific harms that had occurred as a result of engaging in research projects. One organization reported losing a syringe exchange site after increased foot traffic from research recruitment brought the site to neighbors’ attention. More commonly, however, harm took the form of diverting scarce time or resources from the organization’s mission to the researcher’s needs. Many staff expressed frustration that their labor was frequently invisible or disrespected by academic researchers. Even supposedly ‘simple’ tasks, like referring participants to a study or putting up a poster often entailed extra work like explaining the project to participants or seeking out participants whom CBO staff thought might benefit from the study. Other CBO participants described feeling frustrated when research expanded beyond the originally agreed upon protocol, jeopardizing the CBO’s already scarce time, money, space, and human resources; often with very little professional or financial acknowledgment:

*I’m sitting here …wracking my brain coming up with innovative programming on the ground level but you are presenting it as your research and you’re presenting it as, “These are the ideas that we, the researchers, the PhDs came up with.*” *It was not right, it made me feel devalued, and it really sent me through a small episode of trauma because here I am coming into the professional field, I’m still right at the* entry level because I’m fresh out of school and I’m already being burned by researchers, by white researchers. *And I try not to jump on the race card and talk about Tuskegee and all that, but it only reinforces those types of things when you’re actually experiencing it yourself in modern day times*. *Here you are, I’m HIV positive, I’m working in this field, I’m putting myself out there vulnerable, working all types of crazy hours for my community, and here you are and you’re basically using me as a pawn to diffuse information from my community and upstream it to you and you’re not downstreaming* anything to us. *We couldn’t get any additional resources for the program…There was never any opportunities to get any additional funding, any additional professional development, any additional anything other than having to completely reorganize our schedules when they came to town for the purpose of them absorbing research.*

*-Former HIV CBO staff*

Beyond avoiding harm, CBO staff repeatedly emphasized that they felt researchers had an obligation to provide what benefits they could to the organization during their partnership. The nature of this benefit depended on the individual CBO’s needs and preferences. Some staff pushed for academic acknowledgment, both to raise their organization’s profile and to compensate their staff for extra effort. Many spoke about the potential harms and benefits of study data. Disseminating study results in a way that was accessible and useful to the organization was especially important:

*They come and hang out with us. They come to get to know the people we serve with us. We get the data back [...] and we can use the data for our purposes--grant writing, fundraising, setting up new programs, setting up new program sites. We can benefit from that too. We love to hear back from people, how did this go or what did you learn?*

*-Harm Reduction Director*

Other CBO staff emphasized the harm that could come from poorly planned research or study designs that hadn’t involved community members in early stages of. Many expressed concerns about the ways in which ignorant research questions could harm their clients or ability to provide services by increasing stigma, particularly the risk of researchers reinforcing harmful stereotypes about race, class, and substance use due to a lack of cultural competence or humility:

I think the researchers need to interrogate … how their presence might shape the way that people might frame their experience […] it’s like we’re just used to telling white folks our horrible stories. It’s like slave narrative. *It’s kind of like structure our narratives in a way that affirms some of the racist assumptions that might already be present.*

-Black MSM CBO founder and director

### Justice

The notion of justice infused nearly all our conversations. Similar to the ways in which justice is conceived in the Belmont report, these conversations tended to follow two distinct threads: (1) Who is primarily receiving the benefits of a study, and who is primarily shouldering the harms? and (2) Is the institutional knowledge of a CBO treated equally to the academic knowledge of the researcher? CBO staff’s frustrations with academic researchers often resulted from feeling disrespected in situations where either or both questions were at play.

Both professional academic researchers and CBO staff spoke about the unequal distribution of risks and benefits between organizations, clients, and researchers. Many felt that academic researchers shouldered a relatively small amount of the risks arising from the study, compared to the potential for coercion, retraumatization, or stigmatization faced by clients, and the risk CBO’s ran of losing already scarce time and resources with little in the way of remuneration, capacity building, or operational data to show for the experience.

Researchers blow my mind because they just come in with this hubris that they know everything or because they did some study once, that they’re suddenly an expert, which is really fucked up […] we got an email from some fucking organization that they said that they just up and decided that they were going to do a policy paper on decriminalization of prostitution and HIV and they were applying … for sixty thousand dollars and they wanted to know if we’d give them a letter of support…and I was like, “A) Fuck you, B)You guys don’t know anything about this. *We’ve already been working towards this […]We’re already doing this work, you don’t need to go and take sixty thousand dollars out of the potential pockets of sex worker organizations to do this.*

*-Sex Work CBO staff member*

## Discussion

The importance of respectful person-to-person relationships between researchers and community-based organizations is at the heart of our findings. CBO staff repeatedly discussed the role of not only their relationship with the researcher, but the researcher’s willingness to be ‘in relationship’ with their staff, organization, and clients. This willingness to be in relationship strongly influenced CBO staff’s desire to initiate or continue work with a researcher. The researcher’s ability and willingness to be in relationship with the CBO, their clients, and (by extension) the broader community is influenced by their respect for that community and their ability and desire to enact cultural humility to meet the community, CBO, and clients where they are. Willingness to be in relationship with CBOs and community was also the main determining factor as to whether or not an academic’s final impact on the organization – regardless of their intent – actually resulted in at the very least a lack of harm, and ideally in some benefit to the organization. Willingness to be in relationship influenced the researcher’s ability to perceive potential harms and benefits to individual research subjects, the community, and the CBO itself as a distinct entity. The impact of the researcher’s previous actions – or that of a preceding institution or academic researcher—would frequently precede them in a community, dictating whether a CBO decided to engage in a relationship with them again. While our data were collected in North America, these findings broadly align with research conducted internationally, suggesting that these findings are likely not unique to the United States based individuals we spoke with, nor to the specific ‘higher risk’ populations of which the CBOs we spoke with were composed of and/or served (Pratt et al., [35]).

The CBO staff and academic researchers who participated in our study expanded the notions of respect, beneficence, and justice to encompass the relational ethics of researcher-community interactions, beyond the principlist framework most commonly utilized in human subjects research regulations. Study participants emphasized the need to infuse these values throughout the entire research process, not just when considering the rights of individual human subjects who are eligible for enrollment into a study. This emphasis echoes the work of feminist social science methodologists, who have called on researchers to consider the *praxis* of research along with its intended theoretical impact—how the process of conducting research aligns with or attempts dismantle pre-existing systems of oppression (Lather, [36]).

Nearly every CBO participant emphasized ‘respect’ as a holistic ideal which went significantly beyond typical applications of as operationalized by institutional review boards and biomedical ethics training. CBO participants consistently framed their preferences, considerations, and frustrations with academic-CBO partnerships in the language of respect, and academic researchers who had experience partnering with CBOs echoed this emphasis. Study participants had a similarly expansive notion of the idea of justice, encouraging academic researchers to consider both the broader purpose and impact of their study, as well as the fairness with which their organization was being treated.

The expanded notion of respect aligns strongly with the relational framework as it evolved out of feminist calls to consider the positionality of the researcher as well as the research subject, and the importance of considering ‘care’ as a value in biomedical ethics. A key critique of the dominant principlist approach to human subjects research ethics is the way in which it situates the research project within a positivist perspective, tacitly assuming the possibility of researcher objectivity and the centrality of North American ethical standards. As Caroline Criado Perez [37] has compellingly argued, the failure to name the researcher as an actor within the research project also assumes the maleness and whiteness of the academic researcher. While gender did not emerge as a salient theme per se, the gendered dynamics of these relationships should not be ignored, particularly when considering the ways in which relational ethics emerged as a specifically feminist critique of traditional models of bioethics (Sherwin, [38]). The CBO space is frequently coded as one of ‘care’ work, and hence traditionally gendered as more feminine, while academia has historically been a male-dominated field (Steinberg and Jacobs, [39]; Wright et al., [40]). Indeed, although the vast majority of public health trainees at the undergraduate, graduate, and postdoctoral levels (both MD and PhD) are female [U.S. Department of Education [41]], senior researchers are significantly more likely to be white and male (Khan et al., [42]; Lauer et al., [43]).

In the Belmont Report, respect is primarily enacted by allowing individual human subjects the opportunity to consent with autonomy and full knowledge about the project’s potential risks and benefits. The researcher herself is typically absent from this process—albeit often named in the consent sheet as the individual controlling the research project. These Informed consent sheets are typically standardized across a research institution, with language provided and approved by an Institutional Review Board (IRB). The researcher’s role is to simply provide this form and allow the potential human subject to make their decision about participation. Conversely, CBO staff and researchers who had extensive experience working with CBOs centered the practice of respect around the actions and intentions of the researcher. Respect was demonstrated, practiced, and earned in ongoing human relationship between individual members of the study team, organizational staff, and the organization’s clients. Participants did express frustration when research projects expanded beyond the originally agreed upon protocol (analogous in some ways to violating informed consent), but this was secondary to their overwhelming frustration with researchers who did not act with humility, work towards cultural competence, and demonstrate their willingness to be ‘in relationship’ with the organization. This approach to ‘respect’ in human subjects research which centers individual autonomy was essentially anathema to respect as our participants described the process, instead they championed the need for the researcher to step into a dyadic, and dynamic, process of ongoing mutual discovery.

Both CBO and academic research participants repeatedly discussed the researcher’s obligation to not simply minimize harm, but to actively consider how they might create benefits for the CBO within the scope of the research project. While both minimizing harm and maximizing benefit are key aspects of beneficence, the pragmatic difficulty of maximizing benefit for individual human subjects frequently leads researchers to focus ethical concern on the imperative to minimize harm (non-malfeasance) (Mackenzie et al., [44]; Beebeejaun et al., [45]). As many of our participants pointed out, entering into ongoing relationship with CBOs as distinct entities offers researchers the opportunity to move beyond non-malfeisance and identify opportunities for beneficence, both to the community as a whole and to the CBO as a specific institution. Many participants linked this idea of beneficence, not simply non-malfeasance, to the broader notion of justice. To our participants, justice meant acknowledging the expertise that CBO staff brought to a partnership, that the researcher should act with cultural humility, and actively considering where the harms and benefits of the project were accruing. Moreover, several participants linked the notion of justice in academic-CBO partnerships to broader notions of racial, gender, and class justice. The obligation to enact justice in research translated to an obligation to consider social justice in broader society. When working with CBOs, the researcher has an obligation to consider how their own position within these power structures might influence not only their interactions, but also the research questions and approach they utilize. Participants emphasized that when conducting research on – or with – communities that have been made vulnerable by historic and ongoing injustices, researchers must consider not just the theory underpinning their academic research, but the praxis when engaging in the work of data collection. This can manifest in two distinct ways. First, will the benefits of the research-project-as-work be distributed fairly between the CBO and the researcher? In other words, will the tangible benefits which the CBO receives from supporting the research project be comparable to those which the researcher will accrue via publications, grants, conference talks, and opportunities for promotion? Second, will the way in which the research is conducted reinforce or dismantle pre-existing systems of oppression? Is the research structured to demand ‘slave narratives’—data that reinforces stigmatized understandings of communities that have been made vulnerable by historical and ongoing experiences of white supremacy, imperialism, and cisgendered heterosexual patriarchy? Or does the researcher take advantage of available CBO expertise to understand what type of evidence would be most useful to address root causes of health inequality for the community of interest?

Practically, academic researchers function within academic research institutions. As such, their ability to enact many of the suggestions made by study participants can be helped or hindered by institutional flexibility. For example, several CBOs described using volunteer time as a strategy to vet academic researchers. This is a justifiable strategy on the part of the CBOs. However, the practice can also privilege researchers with the financial security and free time to prioritize community volunteer work (i.e., affluent white men who engage in little to no care work). Universities and departments can counter this risk by providing institutional support for time spent relationship building, acknowledging that this may not result in immediate products. Institutional Review Boards (IRBs) and other ethical oversight entities can also create spaces to consider the importance of respectful relationships and tangible benefits to partner community-based organizations. For example, while study committees for the National Institutes of Health (NIH) are asked to comment on ethical concerns in grant proposals, there is no formal function available for questioning the potential burden that a project may place on community partners. Nor are reviewers asked to weigh the distribution of immediate benefits that accrue to investigators versus community organizations as a result of the project. Finally, while IRBs are required to include at least one member who represents “the community,” it has not always been clear which communities are represented by these IRB members or if their ability to advocate for ethical community or CBO involvement in research is appropriate (Klitzman, [46]). Further attention to relationship building and community engagement by ethical oversight entities at universities has the potential to help bridge the gaps between community interests and academic research priorities, conferring greater benefits to both researchers and CBOs.

## Conclusion

The heart of successful public health research and practice is meeting people where they are. Academic researchers often rely heavily on community-based organizations to facilitate those meetings. While ethical research guidelines exist for engaging with individual research subjects, no comparable guidelines exist for working ethically with organizations. Our interviews with leadership and staff at community based organizations across the United States suggest that the same principles of beneficence, justice, and respect can and should be equally applied to these partnerships just as they are to the relationship between researcher and human subject. Researchers who are institutionally enabled to view themselves as individuals in relationship with organizations and their staff, rather than objective scientists executing a protocol and/or contract are more likely to successfully prioritize treating organizations with respect, beneficence, and justice. This in turn will likely lead to higher quality short and long-term research via mutual learning and sustained, long-term community relationships.

## Data Availability

The datasets analyzed for this study are not publicly available, as the nature of qualitative in-depth interviews makes fully de-identified data impossible. Data are available from the corresponding author on reasonable request, with appropriate participant privacy safeguards.

## References

[1] Poteat T, Resiner S, Miller M, Wirtz A (2020). Vulnerability to COVID-19-related harms among transgender women with and without hiv infection in the Eastern and Southern U.S. J Acquir Immune Defic Syndr.

[2] Jashinsky TL, King CL, Kwiat NM, Henry BL, Lockett-Glover A (2021). Disability and COVID-19: impact on workers, intersectionality with race, and inclusion strategies. Career Dev Q.

[3] California Deparment of Public Health (2020). COVID-19 Health equity playbook for communities: Strategies and practices for an equitable reopening and recovery.

[4] Clinical and Translational Science Awards Community Engagement Key Function Committee Task Force on the Principles of Community Engagement. Principles of Community Engagement. Bethesda, MD: National Institutes of Health; 2011. 197 p. Available from: https://www.atsdr.cdc.gov/communityengagement/pdf/PCE_Report_508_FINAL.pdf.

[5] Wallerstein N, Duran B (2010). Community-based participatory research contributions to intervention research: the intersection of science and practice to improve health equity. Am J Public Health.

[6] Key KD, Furr-Holden D, Lewis EY, Cunningham R, Zimmerman MA, Johnson-Lawrence V, Selig S (2019). The continuum of community engagement in research: a roadmap for understanding and assessing progress. Prog Community Health Partnersh.

[7] Wallerstein NB, Duran B (2006). Using community-based participatory research to address health disparities. Health Promot Pract.

[8] Fregonese F (2018). Community involvement in biomedical research conducted in the global health context; what can be done to make it really matter?. BMC Med Ethics..

[9] Minkler M, Wallerstein N. Improving health through community organizing and community building: A health education perspective. In: Minkler M, editor. Community Organizing and Community Building for Health. 2nd ed. Piscataway, NJ: Rutgers University Press. 2008. p. 26-50.

[10] Israel BA, Parker EA, Rowe Z, Salvatore A, Minkler M, López J, Butz A, Mosley A, Coates L, Lambert G, Potito PA (2005). Community-based participatory research: lessons learned from the centers for children’s environmental health and disease prevention research. Environ Health Perspect.

[11] Beste LA, Chen A, Geyer J, Wilson M, Schuttner L, Wheat C, Rojas J, Nelson K, Reddy A. Best practices for an equitable Covid-19 vaccination program. NEJM Catalyst Innovations in Care Delivery. 2021;2(10). 10.1056/CAT.21.0238.

[12] The Nuremberg Code [Internet]. Trials of war criminals before the Nuremberg military tribunals under control council law. 1949;10(2):181-2. Available from: https://portal.abuad.edu.ng/lecturer/documents/1610026743Nuremberg,_Helsinki.pdf.

[13] Association WM (2001). World medical association declaration of helsinki. ethical principles for medical research involving human subjects. Bullet World Health Org.

[14] National Commission for the Protection of Human Subjects of Biomedical and Behavioral Research. The Belmont report: Ethical principles and guidelines for the protection of human subjects of research [Internet]. Bethesda, MD: U.S. Department of Health and Human Services; 1979. 10 p. Available from: https://www.hhs.gov/ohrp/regulations-and-policy/belmont-report/read-the-belmont-report/index.html. 25951677

[15] Hammersley M (2013). On ethical principles for social research. Int J Soc Res Methodol.

[16] Holm S (1995). Not Just Autonomy – The principles of American biomedical ethics. J Med Ethics.

[17] Banks S, Armstrong A, Carter K, Graham H, Hayward P, Henry A, Holland T, Holmes C, Lee A, McNulty A, Moore N, Nayling N, Stokoe A, Strachan A (2013). Everyday ethics in community-based participatory research. Contemporary Soc Sci.

[18] Douglas H (2004). The irreducible complexity of objectivity. Synthese.

[19] Anderson W (2021). The whiteness of bioethics. Journal of Bioethical Inquiry.

[20] Heimer C (2013). “Wicked” ethics: compliance work and the practice of ethics in HIV research. Soc Sci Med.

[21] Etherington K (2007). Ethical research in reflexive relationships. Qual Inq.

[22] Ward L, Gahagan B (2010). Crossing the divide between theory and practice: research and an ethic of care. Ethics and Soc Welfare.

[23] Brugge D, Kole A (2003). A case study of community-based participatory research ethics: the healthy public housing initiative. Sci Eng Ethics.

[24] Flicker S, Travers R, Guta A, McDonald S, Meagher A (2007). Ethical dilemmas in community-based participatory research: recommendations for institutional review boards. J Urban Health.

[25] Mikesell L, Bromley E, Khodyakov D (2013). Ethical community-engaged research: a literature review. Am J Public Health.

[26] Shore N (2006). Re-conceptualizing the Belmont Report: A community-based particpatory research perspective. J Community Pract.

[27] Participants in the Community Engagement and Consent Workshop (2013). Kilifi, Kenya, March 2011: consent and community engagement in diverse research contexts: reviewing and developing research and practice. J Empir Res Hum Res Ethics.

[28] Haraway DJ. Primate Vision: Gender, Race, and Nature in the World of Modern Science. New York & London: Routledge; 1989/1992.

[29] Hill Collins P (2000). Black feminist thought: knowledge, consciousness and the politics of empowerment.

[30] Tebes JK (2005). Community science, philosophy of science, and the practice of research. Am J Community Psychol.

[31] Institute of Medicine (US) Committee on the Social and Behavioral Science Base for HIV/AIDS Prevention and Intervention. Assessing the Social and Behavioral Science Base for HIV/AIDS Prevention and Intervention: Workshop Summary. Washington DC: National Academies Press; 1995. Available from: https://www.ncbi.nlm.nih.gov/books/NBK231446/. 25101470

[32] Gladwin CH. Ethnographic decision tree modeling. Sage University Papers: Qualitative Research Methods Series Vol. 19. 1989.

[33] Creswell JW, Poth CN. Qualitative inquiry and research design: Choosing among five approaches. 4th ed. Los Angeles, CA: Sage. 2018.

[34] VERBI Software. MAXQDA 2022. Berlin: VERBI Software. 2021. Available from: https://www.maxqda.com.

[35] Pratt B, Seshadri T, Srinivas PN (2020). What should community organisations consider when deciding to partner with researchers? a critical reflection on the Zilla Budakattu Girijana Abhivrudhhi Sangha experience in Karnataka. India Health Research Policy and Systems.

[36] Lather P (1986). Research as praxis. Harv Educ Rev.

[37] Perez C. Invisible women: Data bias in a world designed for men. United States: Abrams; 2019.

[38] Sherwin S (2008). Whither bioethics? How feminism can help reorient bioethics. Int J Feminist Approach Bioethics.

[39] Steinberg RJ, Jacobs JA. Pay equity in nonprofit organizations: Making women’s work visible. In: Odendahl TJ and O’Neill M editors. Women and power in the nonprofit sector. San Francisco, CA: Jossey-Bass. 1994. p. 79–120.

[40] Wright HR, Cooper L, Luff P. Women’s ways of working: circumventing the masculine structures operating within and upon the University. Women’s Stud Int Forum. 2017;61:123–31.

[41] U.S. Department of Education Integrated Postsecondary Education Data System, National Center for Education Statistics. Awards/degrees conferred by program (6-digit CIP code), award level, race/ethnicity, and gender: July 1, 2020 to June 30, 2021. 2021. Available from: https://nces.ed.gov/ipeds/datacenter/DataFiles.aspx.

[42] Khan MS, Lakha F, Tan MMJ, Singh SR, Quek RYC, Han E, Tan SM, Haldane V, Gea-Sánchez M, Legido-Quigley H (2019). More talk than action: gender and ethnic diversity in leading public health universities. The Lancet.

[43] Lauer M, Patel K, Rowchowdhury D. RPG and R01-Equivalent Funding and Success Rates by Race-Ethnicity FY2010-FY2021 [Internet]. National Institutes of Health; 2022. 24 p. Available from: https://diversity.nih.gov/sites/coswd/files/images/RPG-by-Race-2-9-22.pdf.

[44] Mackenzie C, McDowell C, Pittaway E (2007). Beyond ‘do no harm’: The challenge of constructing ethical relationships in refugee research. J Refug Stud.

[45] Beebeejaun Y, Durose C, Rees J, Richardson J, Richardson L (2015). Public harm or public value? Towards coproduction in research with communities. Eviron Plann C Gov Policy.

[46] Klitzman R (2012). Institutional review board community members: who are they, what do they do, and whom do they represent?. Acad Med.

